# Surface Plasmon Resonance Based Temperature Sensors in Liquid Environment

**DOI:** 10.3390/s19153354

**Published:** 2019-07-31

**Authors:** Joyce Ibrahim, Mostafa Al Masri, Isabelle Verrier, Thomas Kampfe, Colette Veillas, Frédéric Celle, Serge Cioulachtjian, Frédéric Lefèvre, Yves Jourlin

**Affiliations:** 1 Lyon University, UJM Saint-Etienne, CNRS, Institut d’Optique Graduate School, Laboratoire Hubert Curien UMR 5516, 42000 Saint-Etienne, France; 2 Centre de Thermique de Lyon, UMR 5008, CNRS, INSA, University Lyon, INSA Lyon, F-69621 Villeurbanne, France

**Keywords:** sensor, temperature, surface plasmon resonance

## Abstract

The aim of this work is to measure the temperature variations by analyzing the plasmon signature on a metallic surface that is periodically structured and immersed in a liquid. A change in the temperature of the sample surface induces a modification of the local refractive index leading to a shift of the surface plasmon resonance (SPR) frequency due to the strong interaction between the evanescent electric field and the metallic surface. The experimental set-up used in this study to detect the refractive index changes is based on a metallic grating permitting a direct excitation of a plasmon wave, leading to a high sensibility, high-temperature range and contactless sensor within a very compact and simple device. The experimental set-up demonstrated that SPR could be used as a non-invasive, high-resolution temperature measurement method for metallic surfaces.

## 1. Introduction

The surface temperature at the interface between a solid and a liquid is often difficult to evaluate because the introduction of a probe into the device induces disturbances on the value to be measured [1]. Optical methods can overcome this disadvantage but are not usually adapted to the environment studied. Among them, infrared thermography is a tool that has shown its effectiveness in gaseous media [2,3] but cannot be used for a surface in a liquid environment. The measurement of the surface temperature by detection of a plasmon wave is a promising alternative [4,5,6,7] since the plasmon detects at a nanometric scale any change of the gaseous [8] or liquid medium [9,10] surrounding the surface. In addition, the surface plasmon resonance (SPR) measurement often reaches interesting sensitivities in terms of index variations [6]. Although this technique is well known for applications in the field of sensors in biology [11], chemistry [12] or food industry [13], it has rarely been experimentally studied for the measurement of the temperature of a surface in a liquid environment. Several studies were developed with optical fibers metalized either at their end [14] or along their diameter [15]. The sensitivity of these SPR devices can reach interesting values. As an example in [15], the obtained value is 2.25 nm/°C in the near IR range (around 900 nm). However, the integration of these probes into a high-pressure chamber is difficult to realize.

The work presented here proposes to evaluate the temperature of a plane surface immersed in a liquid in a high-pressure chamber by analyzing the reflected SPR spectrum. To couple the light to a surface plasmon of the metallic surface, the surface itself is microstructured in order to reduce the size of the sensor and to simplify it in comparison with the well-known prism coupling [8]. Even if the configuration with the prism is often more efficient than the grating one [6], it will not be studied here because of the size constraints that make it difficult to implement it within the closed heated chamber filled with liquid at high pressure. Two methods of interrogation of the sensor can be put in place: By angle or in wavelength. We chose the last technique for the same size constraints reasons and also because the sensitivity to temperature is increased in this mode of operation [16]. A preceding article has already shown the possibility of detecting by the same method the condensation of a liquid on a structured surface [17]. In this case, the grating was surrounded by air and the liquid was rising up just below it. A shift in the plasmon resonance wavelength due to a change of the refractive index of the surrounding medium was measured and allowed to study in detail the phenomenon of acetone condensation on the metallic surface. This detection requires an optimization of the position of the resonance wavelength, as well as the amplitude and narrowness of the plasmon signature, which is correlated to the diffraction grating characteristics (material, period, profile and depth) as well as to the environmental conditions (dielectric permittivity, incidence angle). As it is demonstrated in the work presented here, the same principle can be used to measure temperature changes, since the permittivity of the surrounding medium changes with temperature. Here the grating was totally immersed in the liquid. Such a device can be particularly relevant in the energy field for the control and improvement of heat exchange at solid-wall–liquid interfaces where IR thermography is not a suitable technique and SPR using prism coupling is difficult to set-up.

In this paper, SPR at a unidimensionally undulated aluminum surface surrounded by acetone in liquid–vapor equilibrium conditions is first briefly presented, introducing the temperature measurement principle. Then, the modeling and the fabrication of the diffractive structure are studied in detail in order to obtain an SPR of optimized amplitude. Subsequently, the complete device using a chamber with controlled pressure and temperature is presented. Finally, the experimental results are compared to the theoretical predictions, and the performance of the sensor is evaluated, showing that the resolution can reach values comparable to the literature [18], while at the same time opening up the way to measuring setups that are difficult to access with other sensors, notably surfaces immersed in liquids.

## 2. Diffractive Structure and Sensor Behavior

The surface plasmon resonance corresponds to an evanescent wave at the interface of a metal and a dielectric, which is due to a collective excitation of the electrons at the surface of the metal. The dispersion equation in TM polarization of the plasmon mode enables determining its wave-vector $k_{x}\left(\omega \right)$ with the following condition (Equation (1)):

(1) $$k_{x}\left(\omega \right)\approx \frac{\omega }{c}\sqrt{\frac{\varepsilon_{d}\varepsilon^{\prime }{}_{m}}{\varepsilon_{d}+\varepsilon^{\prime }{}_{m}}}\;$$

where ω is the angular frequency of the wave, *c* the light velocity in a vacuum, $\varepsilon_{d}$ the permittivity of the dielectric and $\varepsilon^{\prime }{}_{m}$ the real part of the metal permittivity (supposing the imaginary part is small compared to the real part).

**Figure 1:**
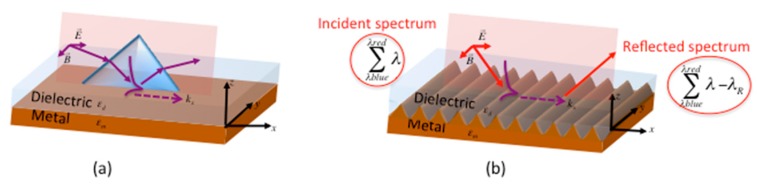
Principle of plasmon coupling at a metal-dielectric interface by (**a**) prism and (**b**) surface structuration.

To excite this plasmon wave, there are two main techniques (Figure 1). The first one is the coupling by a prism: The incident TM wave is totally reflected at the base of the prism and the evanescent wave is coupled to the plasmon mode, which propagates along the metal-dielectric interface with the wave-vector $k_{x}\left(\omega \right)$. The other method is the coupling by a diffraction grating directly printed on the metallic surface: The incident TM wave is coupled to the plasmon mode at the surface of the grating, using the additional k-vector of the grating ($k_{G}=2\pi /\Lambda$).

The plasmon wave exists if the period Λ of the diffraction grating fulfills the synchronism condition (Equation (2)) for the resonance wavelength $\lambda_{R}$, considering the excitation of the plasmon mode with the 1st grating order:

(2) $$2\pi /\Lambda +2\pi /\lambda_{R}n_{d}sin\theta =k_{x}\left(\omega \right),$$

where θ is the incidence angle on the diffraction grating and $n_{d}$ the refractive index of the dielectric. Equations (1) and (2) lead to Equation (3):

(3) $$\lambda_{R}\approx \Lambda \left(\sqrt{\frac{\varepsilon_{d}\varepsilon^{\prime }{}_{m}}{\varepsilon_{d}+\varepsilon^{\prime }{}_{m}}\;}-n_{d}sin\theta \right).$$

Using a polychromatic incident TM wave, the reflected spectrum (zero-order) contains all the spectral components except the one corresponding to the resonance wavelength due to the coupling to the plasmon wave at the grating interface. The surface plasmon resonance is then analyzed via the reflected wavelength spectrum.

As specified in the introduction, the latter method of plasmon coupling by surface structuring is the one chosen for this work mainly for reasons of space constraints. Indeed, to probe the surface inside the closed chamber the beam must pass through a window and the liquid, making large incidence angles as necessary for prism coupling impossible.

### 2.1. Grating Design and Sensor Response at the Operation Point

**Figure 2:**
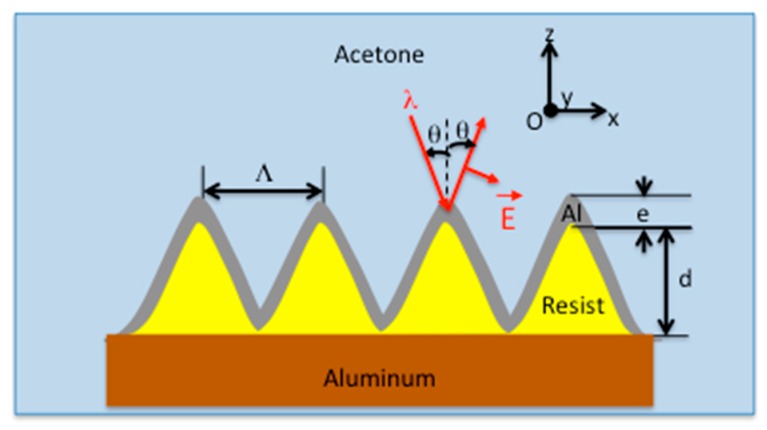
Parameters of the 1D structured sample for plasmonic effect in acetone with e: Aluminum thickness, d: Grating depth, Λ: Grating period, θ: Incidence angle, λ: Wavelength.

The parameters of the 1D diffractive structure (Figure 2) must be chosen to satisfy the resonance Equation (3) for the optimum plasmonic response (maximum amplitude absorption [19,20,21]) for a given wavelength $\lambda_{R}$. The study was first carried out with the sample immersed in a reservoir open to the atmosphere and filled with acetone at room temperature in order to set its parameters at the operating point. In these conditions, acetone is in contact with air at atmospheric pressure and its boiling point is equal to 56 °C.

The material of the structure (substrate and upper layer) is aluminum due to its favorable thermal and plasmonic properties [22]. The sinusoidal diffraction grating is made of photoresist with a period Λ and a depth d. These two parameters have to be defined as well as the thickness e of the aluminum layer on the resist. Finally, the incidence angle θ in acetone is chosen so that the resonance wavelength is in the visible range of the spectrum ($400\;\mathrm{nm}\;<\;\lambda_{R}<\;800\;\mathrm{nm}$).

Simulations of the reflection spectrum were carried out using a commercial program ([23]) based on the Chandezon method, the permittivities of the metal and of the dielectric being respectively $\varepsilon^{\prime }{}_{m}=-51.26$, $\varepsilon_{d}=1.8482$ for the yellow line of sodium $\lambda_{D}=589\;\mathrm{nm}$ at room temperature $T\;=20{\;}^{\circ }$C and at atmospheric pressure.

**Figure 3:**
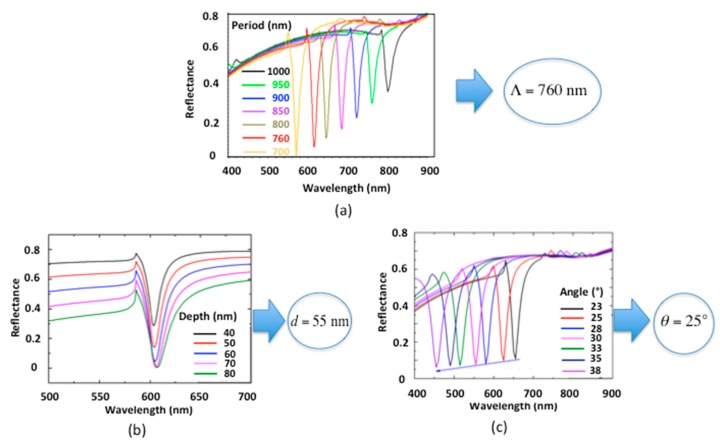
Simulated TM reflection spectra in acetone for: (**a**) Grating periods 700 nm < Λ <1000 nm (aluminum thickness e= 50 nm, grating depth d = 55 nm, incidence angle $\theta =\;25^{\circ }$); (**b**) Different grating depths d (Λ= 760 nm, e = 50 nm, $\theta =\;25^{\circ }$); (**c**) Different incidence angles θ (Λ= 760 nm, e =50 nm, d=55 nm).

In Figure 3a, the reflection spectrum (0th reflected order in TM polarization) was simulated for several periods Λ of the diffraction grating with a smooth undulation d = 55 nm and a moderate incidence angle $\theta \;=\;25^{\circ }$. The metal layer on the resist is chosen thick enough (e = 50 nm) so that no significant transmission occurs. In order to have an operating point in the middle of the visible spectral range, a grating period of Λ = 760 nm was fixed. Once the period was chosen, the influence of the grating depth d was studied. Figure 3b shows that the resonance depth increases with larger values of d but changes only slightly for d > 60 nm. As the best compromise between easy fabrication and optical performance, d = 55 nm was chosen. Finally, the reflectance was studied as a function of the incidence angle θ scanned from $\theta =\;23^{\circ }$ to $\theta =38^{\circ }$ as shown in Figure 3c with Λ= 760 nm, e = 50 nm and d = 55 nm. With an angle of $\theta =25^{\circ }$ in acetone an SPR of sufficient amplitude (55%) at a wavelength close to $\lambda_{R}\;=\;600\;\mathrm{nm}$ in the desired spectral range can be realized. 

As a result, the parameters of the diffractive structure are set as follows: Period Λ= 760 nm, depth d = 55 nm and metal thickness e = 50 nm with an incidence angle onto the grating in acetone of $\theta =25^{\circ }$ corresponding to an incidence angle in air $\theta_{air}=32^{\circ }$ outside the closed chamber.

### 2.2. Simulation of the Plasmonic Response versus Temperature

**Figure 4:**
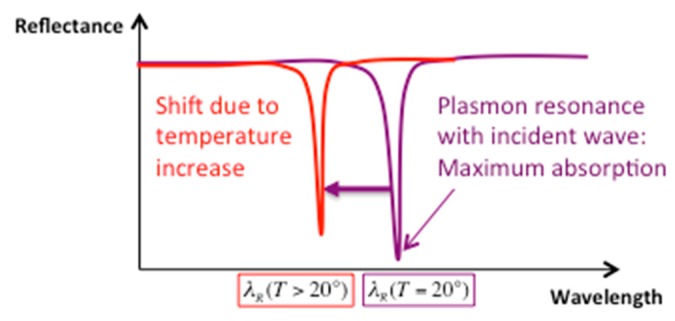
Scheme of reflected spectrum and wavelength resonance shift versus interface temperature.

Once the parameters of the structure set at room temperature $T\;=\;20{\;}^{\circ }C$, the behavior of the resonance versus a temperature change was studied theoretically. The aluminum sample was illuminated through the liquid in TM polarization and the reflected beam was analyzed. The diffraction grating couples the incident light to the surface plasmon wave, allowing a maximum absorption at the resonant wavelength $\lambda_{R}\left(T\;=\;20{\;}^{\circ }C\right)$ that appears as a dip in the reflected spectrum (Figure 4).

**Figure 5:**
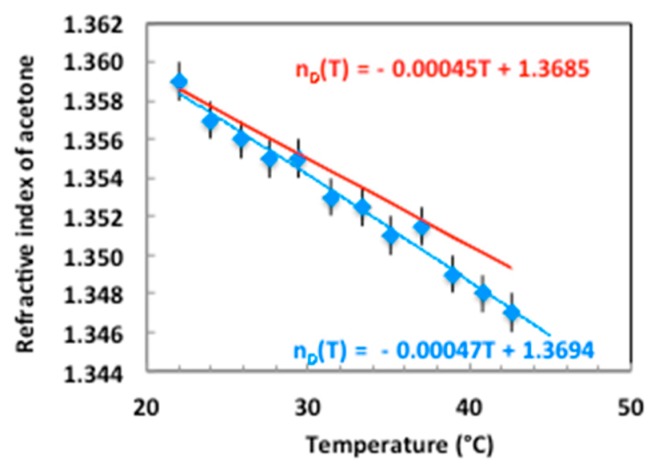
Refractive index of acetone versus temperature—measurements in blue and theoretical curve in red.

When the sample is heated, the permittivity of the medium surrounding the diffractive structure decreases and the resonance shifts to shorter wavelengths ($\lambda_{R}\left(T\;>\;20{\;}^{\circ }C\right)\;<\lambda_{R}\left(T\;=\;20{\;}^{\circ }C\right)$). In the temperature range from $20{\;}^{\circ }C$ to $120{\;}^{\circ }C$, the real part of the aluminum permittivity $\varepsilon^{\prime }{}_{m}$ slightly varies for the yellow line of sodium $\lambda_{D}\;=\;589\;\mathrm{nm}$ ($-51.26\;\leq \;\varepsilon^{\prime }{}_{m}\;\leq \;-50.8978$) whereas the acetone refractive index changes between $20{\;}^{\circ }C$ and $45{\;}^{\circ }C$ as seen in Figure 5 which represents the plots of theoretical [24] and measured (by an Abbe refractometer) refractive index values versus temperature. The mismatch in the slopes of the two curves may be due to both impurities in the acetone used for experiment and uncertainty in dielectric permittivities values.

**Figure 6:**
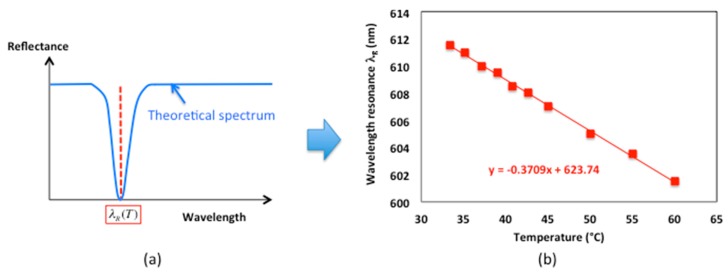
(**a**) Determination of the theoretical resonance wavelength in the spectrum for each temperature; (**b**) Linear dependence of the theoretical resonance wavelength versus temperature.

For each permittivity of the metal and dielectric as well as for each refractive angle in acetone (keeping $\theta_{air}\;=\;32^{\circ }$) at the temperature T, the reflected spectrum was again calculated and the wavelength corresponding to the minimum of reflectance was determined, leading to the plasmon resonance wavelength as a function of the temperature $\lambda_{R}\left(T\right)$ (Figure 6a). For the temperatures $T\;>\;45{\;}^{\circ }C$ above the measurement limit due to the ebullition of acetone (boiling temperature $T_{C}\;=\;56{\;}^{\circ }C$ at P = 1 bar), the values of the refractive index were extrapolated from the measured ones and introduced into the simulation.

As it will be confirmed in the experimental part three where the optical measurements results are presented in detail, the theoretical resonance wavelength varies linearly as a function of the temperature with a slope that will determine the sensitivity of the method (Figure 6b).

## 3. Fabrication of the Sensor and Temperature Measurements

In the previous paragraph, the measurements were obtained at room pressure with fluid acetone being in contact with air. In this paragraph, all the measurements are performed within a tight reservoir, previously emptied with a vacuum pump. The reservoir is filled with acetone in two-phase conditions whose purity is higher than 99.9%. Therefore the saturation pressure inside the reservoir is linked to the saturation temperature by the Clausius–Clapeyron equation.

### 3.1. Fabrication of the Resonant Grating

**Figure 7:**
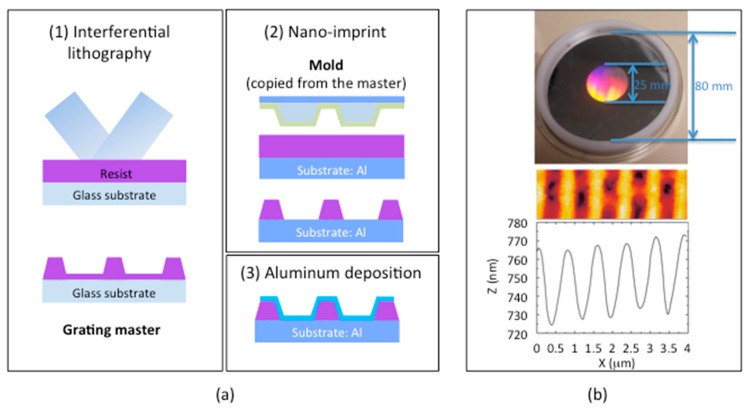
(**a**) Grating fabrication process steps: (1) Interferential photolithography leading to the master; (2) nano-imprint leading to the replication of the mold copied from master; (3) aluminum deposition; (**b**) diffraction grating: Photograph and AFM image.

In order to experimentally verify the theoretical results, the diffraction grating, used as a probe in the SPR device, was manufactured with parameters according to the simulation (Figure 7a). The first step, performed at Hubert Curien laboratory, is laser interference photolithography. The resist layer deposited on the substrate was exposed by an interference fringe system whose interference distance corresponds to the grating period. After development, the diffractive sinusoidal structure of the desired depth was revealed as the grating master. The next step is a nano-imprint process [25] developed by SILSEF company. A polymer was deposited on the aluminum substrate, embossed by a PDMS mold copied from the master diffraction grating and hardened. The grating shape was then printed on the sample. The last step consists in depositing a thin 50 nm layer of aluminum by sputtering. The advantage of this technique is the good reproducibility of the geometrical parameters of the diffraction gratings, once the master is fabricated.

An example of an aluminum diffraction grating is shown in (Figure 7b). The image obtained by Atomic Force Microscopy (AFM) shows that the diffraction grating has a quasi-sinusoidal profile with a period Λ = 760 nm and a depth d ≈ 55 nm close to the optimized structure.

This diffraction grating may then be used as a temperature sensor in the reflectance measurement system described in the following paragraph.

### 3.2. Set-Up for Temperature Surface Measurement via Plasmonic Effect

**Figure 8:**
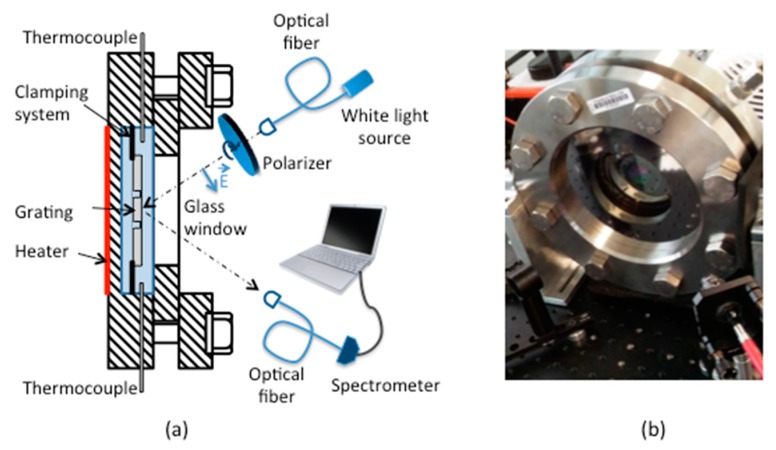
Sensor for surface temperature measurement via a plasmonic response. (**a**) Set-up; (**b**) photograph.

The diffraction grating was placed vertically in a hermetically sealed chamber filled with acetone, which was uniformly heated by a hot plate behind the reservoir ($T\;\leq \;150{\;}^{\circ }C$). An annular groove was made on the back of the sample whose internal diameter corresponds to the diameter of the diffraction grating limiting the heat transfer and thus maintaining an identical temperature throughout the study area. The reservoir was able to withstand a pressure up to 20 Bar (T_sat_ ~ 180 °C). The acetone temperature was controlled by two thermocouples inside the chamber. A collimated halogen source illuminated the diffractive structure on a surface of approximately $50\;{\mathrm{mm}}^{2}$ through the glass window under the desired incidence ($\theta_{air}\;=\;32^{\circ }$) and TM polarization. The plasmon wave was coupled to the grating surface and the reflected spectrum was collected by a collimator of focal length f = 11 mm connected to an optical fiber with a core diameter of 600 μm and to a compact spectrometer and the signal was analyzed subsequently versus the temperature by a dedicated software (Figure 8).

### 3.3. Experimental Spectra

**Figure 9:**
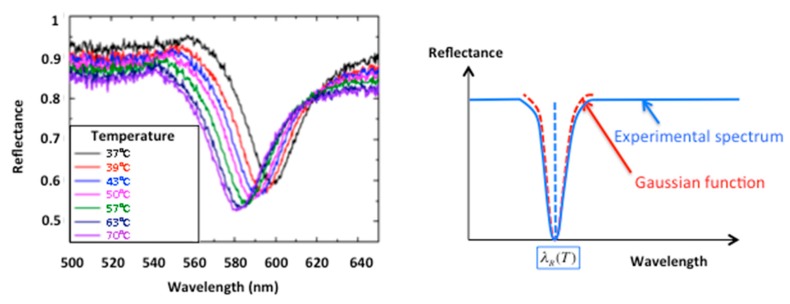
Experimental wavelength spectra for different temperatures and the principle of the resonance position determination.

Figure 9 shows the experimental results obtained at different temperatures from $T\;=\;37{\;}^{\circ }C$ to $T\;=\;70{\;}^{\circ }C$. The diffraction grating was illuminated at its center and the reflected wavelength spectrum was normalized with respect to the reflection on the unstructured surface outside of the grating area. The fiber supports of the collimated source and the spectrometer can be moved vertically in order to record the reference signal above or below the diffraction grating while maintaining the same incidence conditions in the horizontal plane. As determined theoretically in Section 2.2, the resonance shifts to smaller wavelengths as the temperature increases. For each experimental spectrum, the minimum of the signal corresponding to the maximum excitation of the plasmonic wave was determined by adjusting it locally to a Gaussian function and by interpolating the value of the minimum, thus making it possible to find the corresponding plasmon resonance wavelength $\lambda_{R}\left(T\right)$. The Gaussian function, chosen for practical reasons in the used setup, has a bell curve profile similar to that of the theoretically expected Lorentz function and allows the ability to determine the spectral position of the resonance minimum in a sufficiently precise manner.

**Figure 10:**
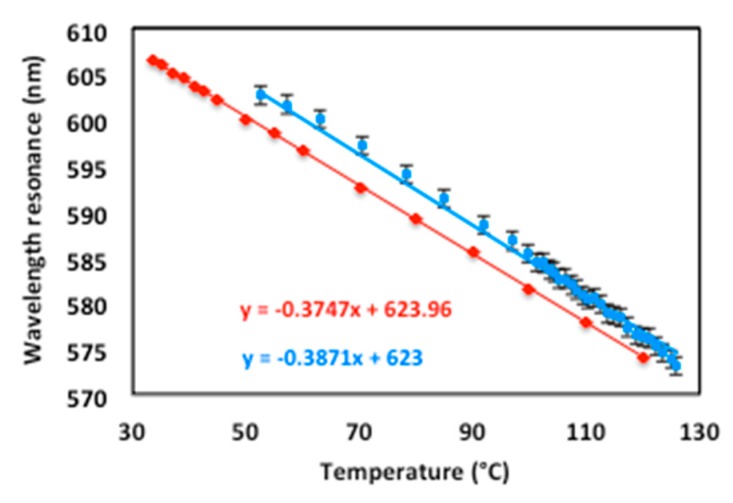
Resonance wavelength $\lambda_{R}\left(T\right)$ versus temperature—experimental data in blue and theoretical in red.

Collecting all the values of plasmon resonance wavelengths as a function of temperature from $T\;=\;54{\;}^{\circ }C$ ($\lambda_{R}\;=\;607\;\mathrm{nm}$) to $T\;=\;125{\;}^{\circ }C$ ($\lambda_{R}\;=\;573\;\mathrm{nm}$) resulted in a linear curve (Figure 10). The sensitivity of the sensor $\frac{\Delta \lambda_{R}\left(T\right)}{\Delta T}\;=\;0.387\;\mathrm{nm}{/}^{\circ }C$ matches well the theoretical one $\frac{\Delta \lambda_{R}\left(T\right)}{\Delta T}\;=\;0.375\;\mathrm{nm}{/}^{\circ }C$ within the measurement uncertainties. The temperature resolution depends on the accuracy of the resonance position determination and on the resolution of the used spectrometer.

**Figure 11:**
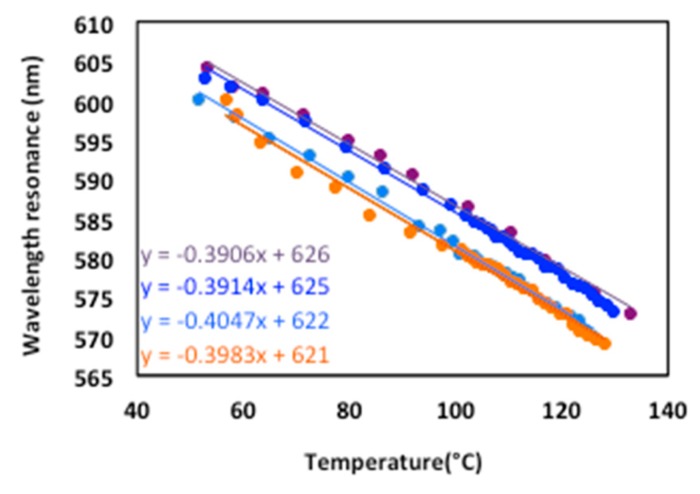
Resonance wavelength $\lambda_{R}\left(T\right)$ versus temperature—measurement repeatability.

Several recordings of the plasmon resonance wavelength as a function of temperature were also made to study the repeatability of the measurements. Each sample was tested four times in increasing heat flux with a rest period of several hours between tests under the same conditions at P = 1.15 bar (Figure 11).

## 4. Discussion and Conclusions

The experimental values of the resonance wavelength are a few nanometers higher than the theoretical ones (Figure 10), which may be due to a slight error on the positioning of the incidence angle relative to that initially fixed. For example, at the operating point ($T\;=\;20{\;}^{\circ }C$, $\varepsilon^{\prime }{}_{m}\;=\;-51.26$ and $\varepsilon_{d}\;=\;1.8482$), the theoretical resonance wavelength is $\lambda_{R}\;=\;616\;\mathrm{nm}$ for $\theta \;=\;25^{\circ }$ and $\lambda_{R}\;=\;624\;\mathrm{nm}$ for $\theta \;=\;24.5^{\circ }$, thus an error of $\Delta \theta \;=\;0.5^{\circ }$ on the incidence angle induces a change of $\Delta \lambda_{R}\;=\;8\;\mathrm{nm}$ on the resonant wavelength.

The sensitivity of this sensor is strongly dependent on the surrounding environment but also on the incidence angle. Indeed, the sensitivity (slope of the red curve in Figure 10) is in theory proportional to the value 1 − sinθ. Decreasing the incidence angle increases the sensitivity of the sensor, for example, an angle of incidence of $\theta \;=\;15^{\circ }$ in acetone instead of $\theta \;=\;25^{\circ }$ makes it possible to multiply its sensitivity by 1.28 while preserving a good quality of the plasmonic response. This angle of $\theta \;=\;15^{\circ }$ is still compatible with the size limits of the device since it corresponds to an incidence in the air of $\theta_{air}\;=\;21^{\circ }$ but reaches the limit of the plasmon resonance wavelength in the visible range ($\lambda_{R}\;=\;772\;\mathrm{nm}$ at $T\;=\;20{\;}^{\circ }C$).

The sensor temperature resolution could be improved by a factor of 10 as well by changing the slit of the spectrometer whose current optical resolution is $\delta \lambda_{R}\;=\;1.5\;\mathrm{nm}$, leading to a minimum expected detectable variation of the temperature of $\delta T\;<\;0.5{\;}^{\circ }C$.

The repeatability of the measurements shows that the sensitivity is varying from $0.40\;\mathrm{nm}{/}^{\circ }C$ to $0.39\;\mathrm{nm}{/}^{\circ }C$, however, the absolute value of the resonant wavelength is substantially greater for the first two tests (blue and purple curves of Figure 11). A very slight change during the tests in the angle of incidence $\theta \;=\;25^{\circ }$ may also be the cause.

To summarize the performance of the sensor used here—the sensor is dedicated to a noninvasive and contactless surface temperature measurement in a liquid environment by using a plasmon resonance. The sensing element is a 1D aluminum diffraction grating that allows direct coupling of the surface plasmon wave. In acetone environment, its sensitivity is $0.387\;\mathrm{nm}{/}^{\circ }C$ in the measuring range from $34{\;}^{\circ }C$ to $125{\;}^{\circ }C$ with a response time of 1 ms. Its performance can be greatly improved by decreasing the incidence angle and by improving the resolution of the spectrometer. Repeatability estimations showed very good stability of the sensor sensitivity, allowing for precise relative temperature measurements. At the same time, the variation of the measured absolute temperature is well above the theoretical resolution limit, which is mainly due to some not sufficiently well-controlled experimental conditions (especially the incidence angle), which can be resolved in the future by integrating the sensor components in a fixed setup.

Finally, even if this device was primarily used for the early detection of the condensation of acetone [17], it is not limited to this particular liquid and as long as the temperature influences significantly the refractive index of the external medium, it can be used with other liquids.
